# Preparation of Smart Materials by Additive Manufacturing Technologies: A Review

**DOI:** 10.3390/ma14216442

**Published:** 2021-10-27

**Authors:** Kunal Mondal, Prabhat Kumar Tripathy

**Affiliations:** 1 Energy & Environment Science & Technology Directorate, Idaho National Laboratory, P.O. Box 1625, Idaho Falls, ID 83415, USA; 2 Nuclear Science & Technology Directorate, Idaho National Laboratory, P.O. Box 1625, Idaho Falls, ID 83415, USA; Prabhat.Tripathy@inl.gov

**Keywords:** additive manufacturing, advanced printing, 3D printing, 4D printing, smart materials

## Abstract

Over the last few decades, advanced manufacturing and additive printing technologies have made incredible inroads into the fields of engineering, transportation, and healthcare. Among additive manufacturing technologies, 3D printing is gradually emerging as a powerful technique owing to a combination of attractive features, such as fast prototyping, fabrication of complex designs/structures, minimization of waste generation, and easy mass customization. Of late, 4D printing has also been initiated, which is the sophisticated version of the 3D printing. It has an extra advantageous feature: retaining shape memory and being able to provide instructions to the printed parts on how to move or adapt under some environmental conditions, such as, water, wind, light, temperature, or other environmental stimuli. This advanced printing utilizes the response of smart manufactured materials, which offer the capability of changing shapes postproduction over application of any forms of energy. The potential application of 4D printing in the biomedical field is huge. Here, the technology could be applied to tissue engineering, medicine, and configuration of smart biomedical devices. Various characteristics of next generation additive printings, namely 3D and 4D printings, and their use in enhancing the manufacturing domain, their development, and some of the applications have been discussed. Special materials with piezoelectric properties and shape-changing characteristics have also been discussed in comparison with conventional material options for additive printing.

## 1. Introduction

Additive manufacturing (AM), often referred to as rapid prototyping or 3D printing in the scientific literature, has evolved as a popular manufacturing technology since the 1980s [1]. In 1986, Chuck Hull recommended the fabrication of three-dimensional (3D) systems via a technology called stereolithography (SLA), which engrossed researchers’ attention and subsequently gave birth to AM technology, also known as 3D printing (or 3DP) [2,3]. Since then, 3D printing has been creating a sound reputation in all of manufacturing, although microstructures fabricated using 3D printing are primarily static in nature [4]. Three-dimensional printing is being widely used in polymer science and technology, space, nuclear, energy storage, biomedicine, and other fields owing to the rapid prototyping capability of 3D products even for complex designs/shapes [5,6,7,8,9]. In the manufacturing sector, 3D printing is mostly used for fabricating complex 3D objects and components of new products in the early stages of development. [10]. Although endowed with many advantageous features, one of the challenges remain in the layer-by-layer printing speed [11] and, precisely because of this reason, 3D printing has not been able to substitute traditional manufacturing methods completely or at least to a significant extent [2].

It was evident that the 3D microstructures manufactured out of smart materials, depending mostly on the functional properties of these materials, can change over time in a prearranged way. This has given birth to a new printing technology by Professor Tibbits in 2013 and introduced to the world as “4D printing” [12]. Four-dimensional printing (or 4DP) was first described with the formula of “4D printing = 3D printing + time”, which denotes the changes in the function, structure, or shape, of 3D printed objects over time [2,13]. This next generation printing uses smart materials with special properties and sophisticated programmable designs to prompt the 3D printed part to change its shape [14]. Given the ongoing development in the AM research and development, the description of 4D printing is expected to become more comprehensive [15,16]. Three-dimensional printing is a predesigned printing of a finished object, whereas 4D printing is the preprogrammed design of a 3D preprinted product into a flexible smart material, tailored with specific properties. The characteristic differences between 3D and 4D printing have been outlined inTable 1 [17].

Compared to 3D printings, 4D printing has many advantages including material flexibility, which enables the exact configuration of material sensitivity with external energy stimuli [18]. Four-dimensional printing can print many 3D parts and objects that 3D printing can’t [19] The color [20], volume [21], and shape [22] of these printed objects can grow or shrink with environmental conditions and stimuli, such as light, water, air and temperature [12]. Four-dimensional printing has been applied to many fields successfully, such as bioprinting where printed biostructures can alter the material functionalities [23].

This review article describes the 3D printing as a representative additive manufacturing technique upon which the discussions on the 4D printing technique are based. The merits and demerits of various printing techniques are discussed. A section on the fabrication of smart materials, including piezoelectric and shape changing materials, by both 3D and 4D printing is also included. Some specific applications of these advanced printings techniques, their current trend and future perspectives for this exciting new field have also been highlighted. 

## 2. Advanced Printing Strategies

### 2.1. Three-Dimensional Printing (3DP)

The 3D printing method fabricates a 3D object from a CAD model, typically by sequentially adding thin material layers between 16 to 180 microns or more to create an object in a layer by layer (LBL) approach. This is also broadly known as additive manufacturing, and differs from conventional fabrication process, where the fabricated material is detached from an object via the regular casting and/or forging methods. To date, many reviews have been published on this topic and a few of them have provided interesting discussions over the advantages, challenges and recent developments in the 3D printing technologies depending on specific applications [24,25,26]. 

Recent 3D printing growth and expansion have not only focused on the refining of the quality of printed objects but also on the size of yields, initiating an entire spectrum of new opportunities for the printing technology. Three-dimensional printing is becoming a stirring technology: from merely being a means of rapid prototyping to an economically feasible option for mass production of materials by the development of printing competences, in both the size and complexity of end products.

**Figure 1:**
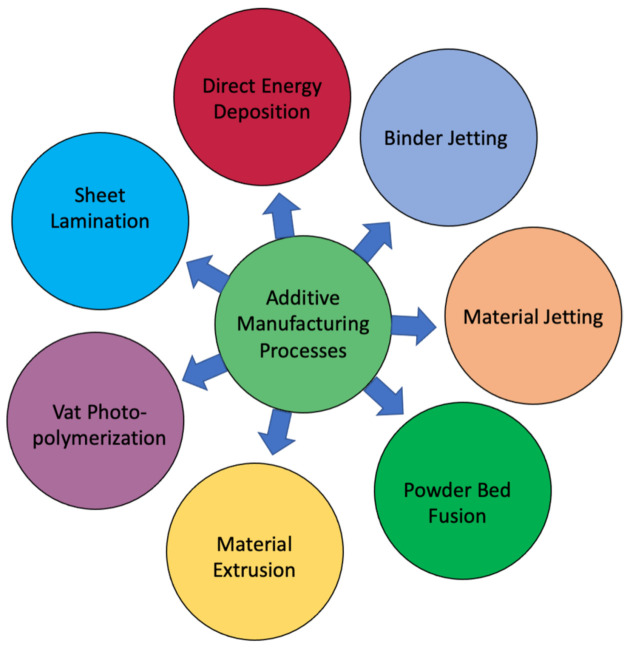
Classification of a typical AM process.

There are three main stages in 3D printing process. The first step is the groundwork just before actual printing, when a 3D CAD file of the object is created. In the next step, the material with required specific properties is chosen, and then the actual printing starts. The printing materials include metals, polymers, ceramics, resins, sand, glass, textiles, biomaterials, and even food. Most of these materials allow adequate allowances to attain precise control over the printed objects. However, some materials, for instance glass, are not easily amenable to precise control of fabrication parameters yet and as a result intense research and development activities are still being pursued to achieve desire control during printing [27]. The final stage is the finishing step which requires the matching of specific instrumentation skills with that of the properties of the functional materials. Once an object is first printed, mostly it cannot be ready to use until it has been cured, lacquered, sanded, or painted to finish it as envisioned. Figure 1 describes various techniques of a typical AM processes, used for printing polymeric, metallic and ceramic components.

**Figure 2:**
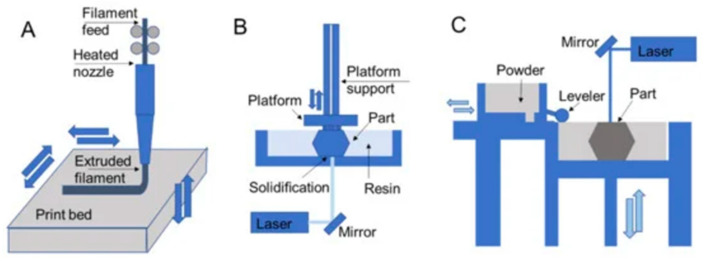
Three-dimensional printing schematics for (**A**) fused deposition modeling, (**B**) stereolithography, and (**C**) selective laser sintering that are representative of extrusion, resin, and powder processes, respectively. Reprinted with permission from Ref [42] Copyright 2020 by the authors and license MDPI, Basel, Switzerland.

**Figure 3:**
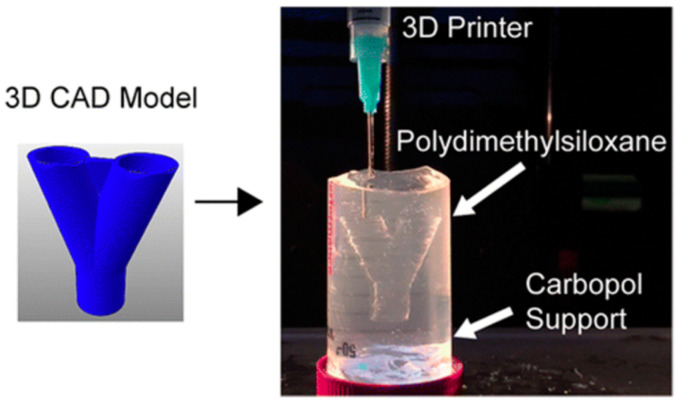
3DP of hydrophobic polydimethylsiloxane prepolymers inside a hydrophilic Carbopol gel supported by a freeform reversible embedding. Reprinted with permission from Ref [43], Copyright 2016, American Chemical Society.

Polymer 3D printing techniques incorporate resin-, powder-based, and extrusion processes (Figure 2) [28,29,30,31,32,33]. Each of these processing step facilitates the layer-by-layer deposition of materials to build the 3D objects and parts [34]. Figure 3 shows that with the recent advances in polymer 3D printing, complex and non-Newtonian polymers, such as polydimethylsiloxane (PDMS), can be used for complex shape building. 

Liquid deposition modeling (LDM) has also been familiarized as a promising manufacturing technology for 3D printing of wood. Wood specimens were 3D printed via paste-like suspensions in aqueous medium [35]. Physical properties in the LDM printed objects were influenced by wood particle size and binder/water ratio. In addition, there are some reports about few new techniques, e.g., volumetric additive manufacturing [36], implosion fabrication [37], and two-photon lithography [38,39]. Table 2 shows comparison of the different types of 3D printing technologies which are mostly used [40,41].

**Figure 4:**
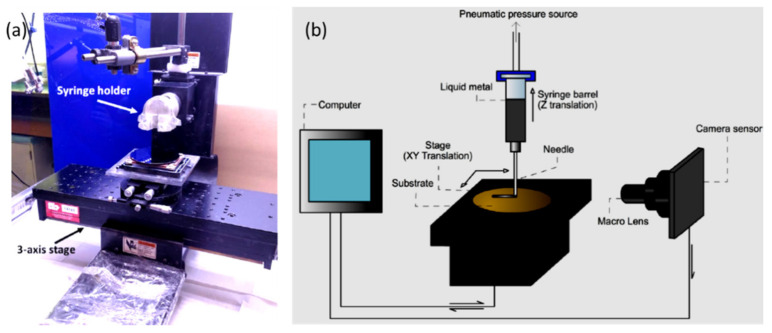
(**a**) Digital photograph exhibiting the custom print stage for direct printing of liquid metal contacts (left) and (**b**) a diagram to explain the 3D printing operation. Reprinted with permission from ref. [47] Copyright (2018) American Chemical Society.

Although metals are the most interesting option as material for 3D printing, there are several other unconventional materials that have also been successfully printed [44,45,46]. Liquid metal is one of them. For instance, recently Andrews et al. [47] reported a direct writing of liquid metal onto SiO_2_-coated carbon nanotube (CNT)/silicon surface using a custom-made 3D printer (Figure 4). They could use liquid metal as conducting ink and explore it as both source and drain contact for a CNT thin-film transistor. He et al. [48] broadly discussed 3D printing of hydrogel and after a series of experiments they concluded that air pressure, feeding rate and printing distance are the most important parameters which affect the overall printing quality. They have also determined that the printing resolution is impacted by the diffusion and fusion of the bio inks. The hydrogel was made from a mixture of sodium alginate and gelatin. The blend was extrusion printed on a substrate at a low temperature to ensure the solidification of gelatin and preserve the bioarchitectures. After the printing, the assemblies were dipped in a CaCl_2_ solution to facilitate the crosslinking of sodium alginate and attain the cell-laden hydrogel structures.

In addition to some of the advantages of 3D printing, such as production speed, flexibility in design and cost factors, there lies a number of challenges too. These include energy-intensive nature of the printing process, problems associated with mass manufacturing of products, and limited availability of ink material for printing. However, for small production runs, and prototyping, a 3D printer is often a massively better manufacturing option than the conventional ones.

### 2.2. Four-Dimensional Printing (4DP)

**Figure 5:**
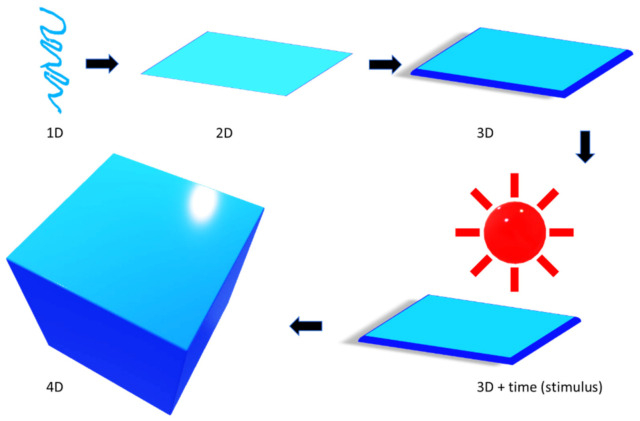
A simplified illustration of the concept of 4D printing.

Figure 5 describes how a 3D printing technique can be transformed to its next higher version (4D).

As the Figure 5 suggests, 4D printing can be envisioned as an advanced printing method where a 3D printed structure alters itself (as shown in Figure 5 with light activation) into a different object with the effect of external energy input, such as heat, light or other available environmental stimuli [49,50,51,52]. Four-dimensional printing is programmed to alter a 3D printed structure, in terms of its shape, property, and functionality [53,54,55,56,57,58]. It is endowed with properties, such as self-assembly, self-repairability, and multifunctionality. It is time-dependent and foreseeable, but independent of the selection of ingredients and choice of the printer. Such an evolutionary characteristic depends mainly on the choice of appropriate smart functional materials in the 3D space. There are at least two stable conditions in a 4D printed objects, and the structure can change from one state to another under the influence of appropriate stimulus. Four-dimensional printing demonstrates several benefits over 3D printing in quite a few aspects [59].

**Figure 6:**
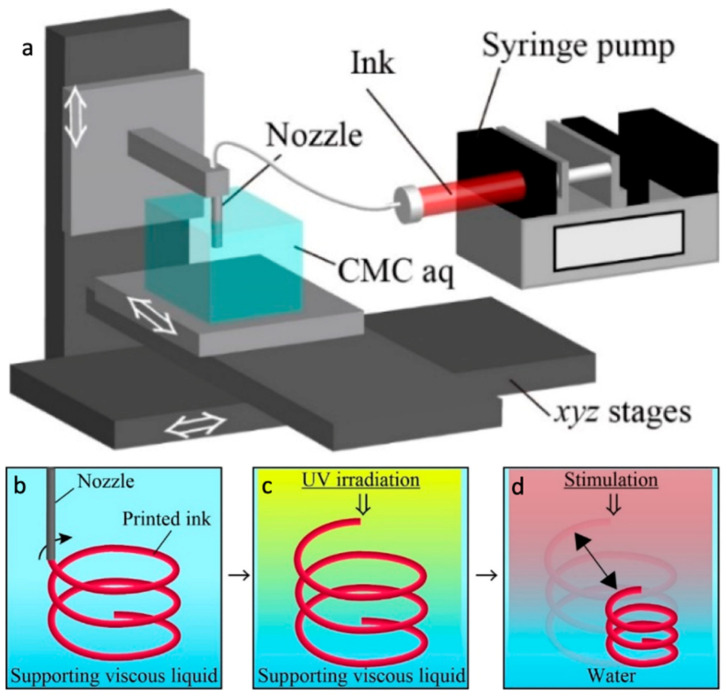
(**a**) Schematic diagram of a 4DP system. It is showing that a nozzle is secured on a *z* stage, and a vessel of carboxymethyl cellulose aq (CMC aq) is secured on *xy* stages. By stirring the *xyz* stages, the pre-gel ink is deposited through the nozzle into CMC aq using a syringe pump. Concept for printing 4D object with internal gaps. (**b**) Pre-gel monomer ink is deposited into auxiliary viscous liquid to print a 3D ink design. (**c**) The written ink is subjected to UV light and is polymerized to acquire a 3D hydrogel assembly. (**d**) Afterward substituting the auxiliary liquid into water, the polymerized 3D hydrogel assembly collapses in reaction to UV light. Reprinted with permission from reference [61], Copyright 2020 by the authors and license MDPI, Basel, Switzerland.

Presently, fused deposition modelling (FDM); stereolithographic apparatus (SLA); selective laser sintering (SLS); and polyjet AM technologies are in use in 4D printing [17]. A 4D printed object can be thought of as an offspring from the marriage between smart material and a 3D printer. It can be developed by exposure to external stimulus through a direct or indirect interaction mechanism, and sometimes mathematical modeling is required for the design of the distribution of multiple materials in the structure [60]. A 4D printing set up is shown in Figure 6a along with the concept of fabricating a 4D structure with internal gaps (Figure 6b–d).

**Figure 7:**
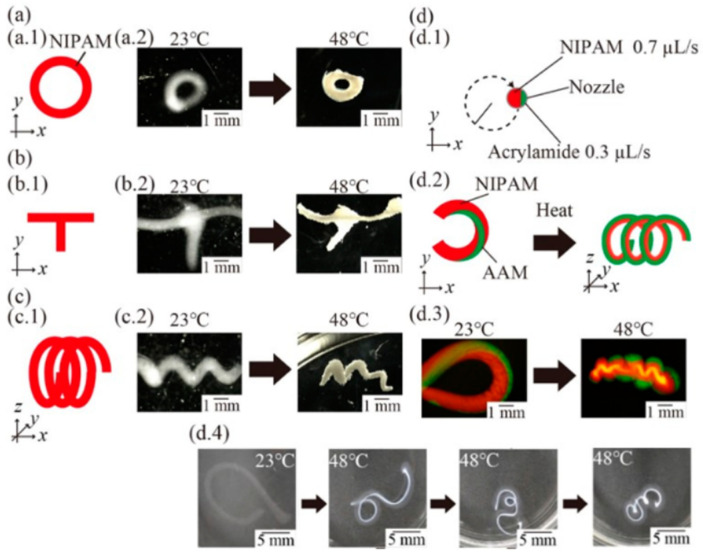
A validation of a 4D printing process. (**a**) Schematic presentation of printed structures and heated structures with a rounded pattern. (**b**) Schematic presentation and images of a printed T-shaped structure and a heated T-shaped structure with a cross point. (**c**) Schematic presentation of a printed spring structure and heated spring structure with an internal gap. (**d**) Schematic presentation of printing a C-shaped structure with multihydrogels (**d.1**) and its 3D shape changing from the C-shaped structure to the spring-shaped structure. (**d.2**) Fluorescence images of the fabricated C-shaped structure (**d.3**) and the transformed spring structure obtained by heating. (**d.4**) Time-lapse images of the C-shaped structure. Reprinted with permission from reference [61], Copyright 2020 by the authors and license MDPI, Basel, Switzerland.

**Figure 8:**
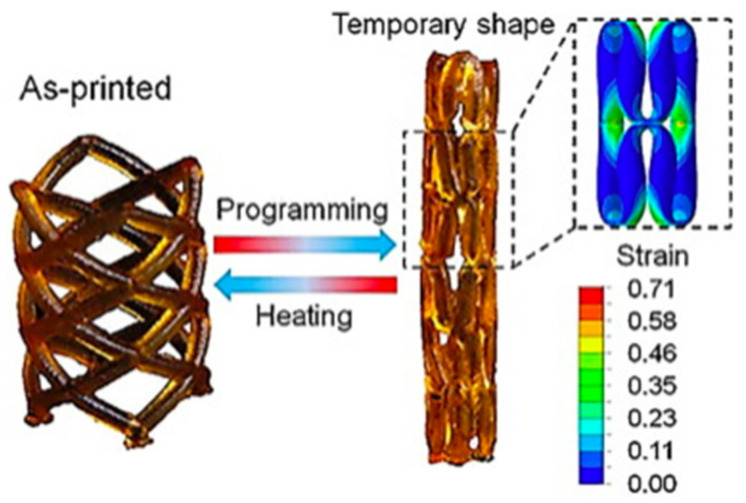
A 4D-printed, thermoresponsive stent which can reversibly change its diameter and height. Reprinted with permission from reference [73], Copyright 2016, Nature.

Three-dimensional printing is a precursor to the fabrication of multimaterial 4D structures, especially with a simple geometry. Typically, a 4D printed structure is shaped by combining several materials into a one-time printed single structure [62]. The variances in material properties, such as thermal expansion coefficient, swelling ratio, modulus etc. led to the anticipated shape-changing effects. A suitable stimulus is also essential to activate the property/shape/functionality variations in the printed object. The selection of the stimulus hinge on the demands of the specific application and the stimuli that have been used in 4D printing thus far consist of heat, light, water, or a combination of the three [63,64,65,66,67,68,69]. Smart or stimulus-responsive material is the other most critical component of 4D printing. The ability of these materials is well-defined by material properties: multifunctional, smart decision making, self-sensing, sensitivity, shape memory, self-adaptability, and self-repair [70,71,72]. A demonstration of a 4DP method is shown in Figure 7 where variations of 4D structures were fabricated to demonstrate the effectiveness of the method. The proposed method allows the printing of complex 4D multi-hydrogel structures with cross points and internal gaps. Figure 8 describes a thermo-responsive, shape memory polymer (SMP)-based, 4D-printed cardiovascular stent, fabricated by Ge et al. [73]. Stents, being vital biomedical devices to enlarge the human vessels, have been at the center of many research studies [74,75].

## 3. Available Smart Material Option for Printing

For any manufacturing technique, including AM, the ink feedstock must be compatible with the printing process (e.g., particle, liquid, powder, wire, sheet). For example, in stereolithography, the feedstock must be a liquid phase thermoset polymeric monomer that crosslinks upon exposer to the appropriate light. Finally, the material must show suitable physicochemical properties to effectively accomplish in the specified application. For the most rigorous uses, printed AM products are typically postprocessed to achieve desirable microstructure, appropriate mechanical properties, with finished surfaces.

**Figure 9:**
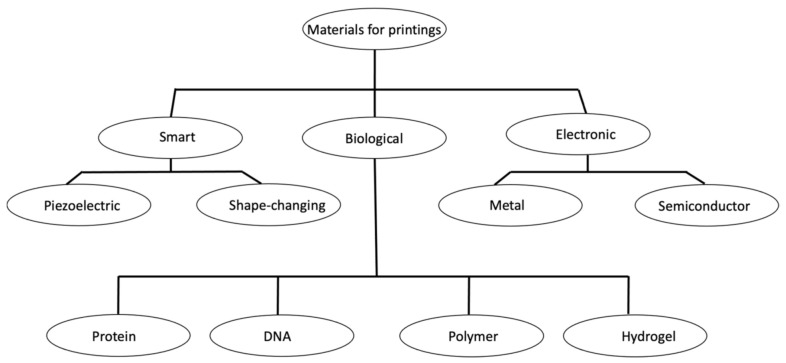
Classification of printable materials that can be printed in advanced printing technologies.

The available materials that can be printed using advanced manufacturing techniques have turned out to be more diverse owing to an advancement in printing knowledge and machineries. These materials can be categorized into three broad areas, such as electronic, biological, and smart materials. The typical used materials include metal, polymer, proteins, DNA, nanowires, and nanotubes. The classification of the printable materials, based on their applications, is shown in Figure 9. Table 3 summarizes these printing materials along with the respective printing strategies and potential applications for an informal comparison.

### 3.1. Smart Materials

Stimulus-responsive materials that alter their shape, size or functional properties under certain stimuli such as solvent, pH, temperature, electricity, light, etc. are known as smart materials [70,76,77]. In other words, smart materials are materials that respond to changes in their environment and then undergo a material property change. These property changes can be leveraged to create an actuator/sensor from the materials without the requirement of any additional control and/or electronics. The marriage of AM with smart materials has given birth to an intelligent research area, known as 4D printing [78]. Even if advanced printing is a newly emerging area, wide-ranging investigations are currently being performed in multiple fields. Present focus on development of smart materials for high-resolution printing does primarily consist of piezoelectric, and shape-changing materials.

#### 3.1.1. Piezoelectric Materials

Piezoelectric materials convert mechanical energy (such as stress) into electrical energy and vice versa. They offer a wide range of utility and can be used as actuators (provide a voltage to create motion), sensors, such as many accelerometers, acoustic imaging, high-resolution 4D printing for the fabrication of smart micro/nano devices such as biosensors and energy harvesters since the charge generated from motion can be harvested and stored. Common applications for piezomaterials are in BBQ igniters and actuators for inkjet printer heads.

**Figure 10:**
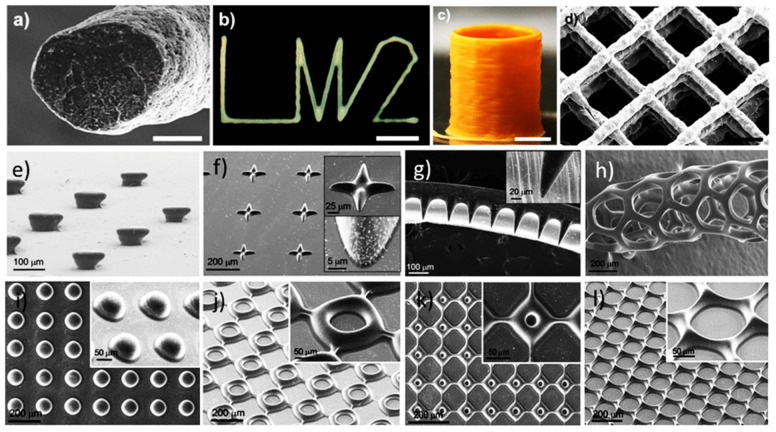
Structures fabricated via photopolymerization using piezoelectric nanocomposite solution. (**a**) SEM image showing the cross-sectional view of a 1D microfiber (scale bar = 25 μm), (**b**) Optical micrograph of a 2D structure as “LM2” (just a n abbreviation; scale bar = 2.5 mm), (**c**) Digital photograph showing inclined side view of a 2.5 D 70layered cylinder (scale bar = 2 mm), and (**d**) SEM image of inclined top-view of a 3D 9-layered scaffold (scale bar = 0.5 mm). Reprinted from reference [80] with the permission of American Chemical Society, Copyright 2014. Three-dimensional structures printed by DOPsL technique including (**e**) a mushroom-like array, (**f**) a cross array, and (**g**) a tapered cantilever array (dark region, cantilever; light region, support). (**h**) Microtubule structure happened by emancipating a honeycomb array from the substrate. The film rolls up after releasing because of small stress gradients in the film. Assembly of piezoelectric microstructures 3D printed using a custom-made DPP printer attached with a 365 nm light emitting diode (LED) as light source counting (**i**) array of dots, (**j**,**k**) square arrays with various sized void spaces, and (**l**) a honeycomb array structure. Reprinted from [81] with the permission of American Chemical Society, Copyright 2014.

Polyvinylidene fluoride (PVDF) is an imperative piezoelectric material and has been used for the high-resolution advanced printings of smart devices. Kim et al. [79] fabricated piezoelectric films from polyvinylidene fluoride (PVDF) polymer using integrated fused deposition modeling (FDM)-based 3D printing technique followed by a corona poling method. They have reported that β-phase of the PVDF undergoes transformation over FDM 3D printing, where a high electric voltage was applied via a corona poling between the printer’s nozzle and the conducting substrate. Bodkhe et al. [80] employed a vat photopolymerization technique to print smart 3D structures with superior dielectric and piezoelectric properties. As shown in Figure 10a–d, piezoelectric/polymer composites of BaTiO_3_ nanoparticles and PVDF polymer were printed in fibrous form, with a size of ~60 μm and stacked together to create a ready-to-use millimeter scale 3D contact sensor which confirmed a maximum voltage output of 4 V upon finger tapping.

Kim et al. [81] used dynamic optical projection stereolithography (DOPsL) technique to fabricate various 3D structures including a random mushroom-like array that has a slighter base area compared to the top, a cross array with a lower center and round edges, and a tapered cantilever array (as shown in Figure 10e–g). Creating structures with complex void regions that are layered on top of each other or features that are hollowed out will require more sophisticated photopolymerization techniques. If the DOPsL printing performed on a substrate and the interactions of the substrate with the photopolymer composite is not strong enough (e.g., hydrophobic surface), the printed object can ride up to form higher order structures. The microtube shown in Figure 10h is one such example where after polymerization the object is detached from the substrate, which rides up into a well-defined tubular structure.

Kim’s group has also employed microscale digital projection printing (DPP) to photopolymerize piezoelectric nanoparticle (barium titanate (BaTiO_3_, BTO)) –polymer colloidal suspensions into predesigned 2D or 3D assemblies in <2 s, shown in Figure 10i–l. Under light irradiation, the chemical cross-linking happens between the polymers and the surface functional groups on the piezoelectric nanoparticles, which binds the nanoparticles to polymer mainstays. This straight linkage of piezoelectric nanoparticles to the flexible polymer matrix improves the piezoelectric output of the composite films by proficiently focusing mechanical stress to the piezoelectric crystals. The structures and arrays fabricated in Figure 10e–l depend on the single photon absorption phenomenon to catalyze the chemical cross-linking process.

#### 3.1.2. Shape-Changing Materials

High-resolution smart assemblies fabricated by advanced printing out of shape-changing materials can shift their shapes/sizes or properties with time, which brands it thinkable of precisely control microscopic shape variations and offers novel way of potential applications in soft robotics, biomedical devices, drug delivery, and microscopic biomimetics. For instance, Kim et al. [82] have demonstrated 3D printing of shape changing materials of ferromagnetic domains. The composite ink for 3D printing contains a magnetizable microparticles of neodymium–iron–boron (NdFeB) alloy and fumed silica nanoparticles impregnated in a silicone polymeric network containing a silicone catalyst and a crosslinker. Furthermore, they developed a model to envisage the revolution of complex 3D-printed structures with programmed ferromagnetic domains experiencing magnetic fields. Ge et al. [49] fabricated lamina composites by direct 3D printing of a programmable glassy shape memory fibers within an elastomeric matrix. Glassy fibers in the size range of 32–64 μm showed shape-changing behaviors when the applied temperature exceeded their glass transition temperature (Tg). This printing approach can be termed as 4D printing, and the printed assemblies can be thermomechanical programmed to form various complex shapes. Wang et al. [83] prepared a shape–color dual responsive composite material using polylactic acid (a shape-memory polymer) and thermochromic pigments. With the smart composites and an FDM 3D printer, shape–color double-responsive advanced printing could be achieved.

**Figure 11:**
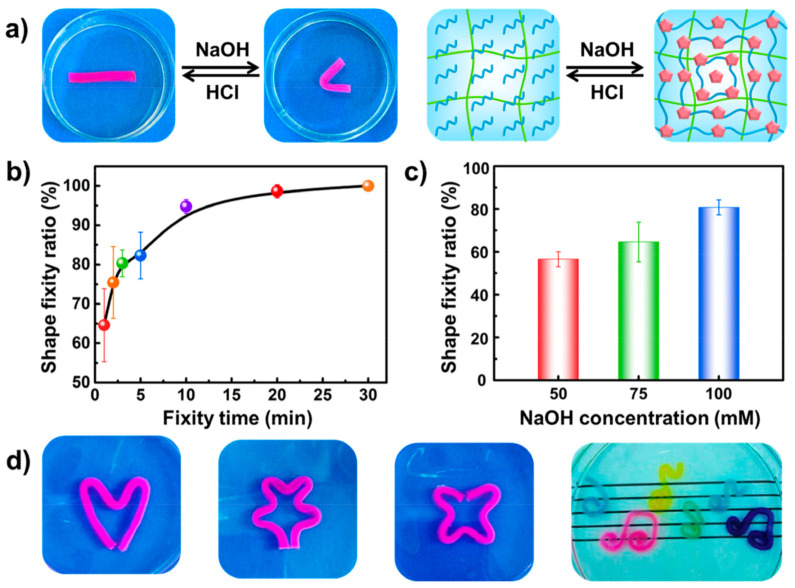
(**a**) The shape memory performance and mechanism founded on chitosan physical microcrystalline crosslink. (**b**) Change in the shape fixity ratios of the PAAm-CS hydrogels as a function of the fixity time in NaOH solution (75 mM). (**c**) Change in the shape fixity ratios as a function of concentration of NaOH solution with a shape fixity time of 1 min. (**d**) Pictures of the more complex transient shapes of PAAm-CS hydrogel secured by NaOH (75 mM). Reprinted with permission from reference [84], Copyright 2017 by the authors and license MDPI, Basel, Switzerland.

Shape memory hydrogels are an irreplaceable class of smart materials. They have the unique capability to retrieve to the initial shapes from transient shapes triggered by external energy stimuli [84]. Thus, shape memory hydrogels have attracted increasing consideration from a diverse range of fields, such as drug release, textiles, smart actuators, and aerospace, etc. Figure 11a–d schematically describes the shape changing capability as depicted in Figure 5, a straight stripe sample was deformed into a “U” when immersed in NaOH solution and recovered its original straight shape once transferred into HCl solution since the microcrystalline physical crosslink junctions destroyed and the hydrogel recovered.

Shape and the memory hydrogels have also been explored for advanced printing as a shape-changing material. Printed hydrogels display high durability and stretchability like biological tissues, which can survive physiological mechanical pressures. Hong et al. [85] demonstrated a high-resolution 4D printing method for the fabrication of extremely deformable tough structure from a combination of poly (ethylene glycol) and sodium alginate-nanoclay hydrogel. The 3D printed assemblies comprised of an interpenetrating network which could be stretched up to 300% of its actual length and then recover to its original shape. Lately, a group of researchers from Rutgers University and New Jersey Institute of Technology [86] have jointly conceived a smart 4D printing process for a shape changing gel that could lead to the progress of printing of “living” assemblies in human organs and tissues, targeted delivery of drugs, and soft robots. The advanced printing method they used contained micro-stereolithographic printing of a 3D shape with a hydrogel material that smartly changed shape with temperatures over time.

Shape-changing biomaterials have advanced high-resolution printing, due to their properties in response to chemical signals. For example, Ling et al. [87] employed a two-photon photolithography technique to print three-dimensional bovine serum albumin (BSA) protein-hydrogel microarchitectures in the size range of 10–30 µm with programmable shape changing features. The high accuracy directional sensitivity of printed 3D structures was attained by controlling the cross-linking density of BSA at the nanoscale. Sun et al. [88] prepared a photo crosslinked protein network with BSA protein and a photosensitizer methylene blue and employed it to print micro-/nanoscale features by directed energy deposition technique. It was also reported that the pH-active materials could be printed onto protein microlenses with diameters in the range 1–100 μm, unveiling a fast and reversible swelling-to-shrinking response.

Shape memory polymers (SMPs) are similar to shape memory alloys (SMAs) except the obvious fact they are made from a polymer matrix. They possess much greater recoverable strains than the shape memory alloys, but typically under lower forces. Morphing structures has been the area of greatest interest, to date, for these SMPs.

### 3.2. Other Materials

In addition to metal and semiconductors, other pure and functional materials, such as carbon and its composites, zinc, lithium ions, and ruthenates have been employed for the AM based printings to yield features with high resolutions. As an important class of nonmetallic conductive materials, carbon-based materials, for instance graphene, CNTs, carbon black, and carbon nanofiber, have been explored recently by making composites with polymers and semiconductor materials due to their excellent conductivity and interesting electronic configurations [89]. For instance, aerosol jet printed single-walled carbon nanotube (SWCNT) networks were used for thin film transistors owing to their high electron and hole mobilities [90]. Ink was prepared by blending multiwall carbon nanotubes (MWCNTs) and polystyrene sulfonate (PSS) for electrohydrodynamic printer to obtain source and drain electrodes for an extremely reliable organic field-effect transistor (OFET) [91]. By tuning the rheological properties, a low-cost suspension of carbon black particles in the form of carbon conductive grease was used as ink to 3D print highly stretchable strain sensors [92]. Carbon black particles and polycaprolactone were mixed to form a thermoplastic conducting ink and 3D printed to fabricate a range of functional electronic sensors from flexible strain sensors to piezoresistive sensors [93]. Park et al. [94] reported electrohydrodynamic jet printing to fabricate high resolution complex patterns using a variety of inks ranging from nonconducting and conducting polymers, to single-walled carbon nanotubes, to solution suspensions of silicon nanoparticles and rods. Fused deposition modeling (FDM) based 3D printing was used to print a composite conductive ink of polylactic acid (PLA) and reduced graphene oxide (RGO) by Zhang et al. [95] and a conductivity of 600 S/cm has been achieved. However, stability and conductivity of the printed carbon-based composite materials are still vulnerable because of the use of nonconducting polymers and their poor dispersity in the composite matrix.

Zinc and lithium are well known metals used for energy storage devices. It was premeditated that advanced printing of these materials could possibly increase energy density by allowing competent use of the limited 3D space within the devices. For example, 3D printed Li_4_Ti_5_O_12_ (LTO) anode and LiFePO_4_ (LFP) cathode in an interdigitated lithium-ion microbattery provides a high areal energy density as well as high power density [96]. Since there was a need for conductive additives in the electrodes, research was performed to solve this challenge and graphene oxide has been added. Increased cycling performance with high specific capacities was reported in addition to high energy and power densities [97].

Apart from lithium-ion batteries, zinc–silver batteries have shown promising results with high specific energies and offers a high-power density [98,99]. For instance, a planar 2D fully printed silver–zinc battery has been reported by Braam et al. [100] Ho et al. [101] fabricated microbattery electrodes from alkaline Zn-Ag which was directly deposited onto substrates by using super ink jet printing technique and the printed electrodes demonstrate enhanced capacity. The effect of ink formulation in the screen-printed electrodes and electrode geometry design has been studied by Saidi et al. on the electrochemical performance of a Zn/MnO_2_ alkaline battery [102]. Very recently, an epidermal alkaline rechargeable nontoxic screen printed Ag–Zn tattoo battery for body-worn electronics has been reported by Wang et al. [103].

Some of the other types of smart materials include shape memory metallic alloys (nickel-titanium), magnetostrictive materials, electroactive polymers and bicomponent fibers. Similar to piezoelectric materials, magnetostrictive materials respond to changes in magnetic fields and can perform as actuators, or sensors if deformed. While they can work well, they exhibit large hysteresis losses, which must be compensated when using these materials for sensor applications. Electroactive polymers are of many forms and some of them are still being refined. They have great potential as the flexibility of how they can be used to provide advantages over some of the metals and ceramics. Typical applications include energy harvesting and sensing. However, some researchers are also looking at developing high voltage, low current actuators out of these materials. As the name suggests, bicomponent fibers consist of two different materials and are coextruded together to enable shape change depending on ambient temperature. These fibers can enable smart clothing that can change thermal properties based on the environment.

## 4. Applications and Trends

**Figure 12:**
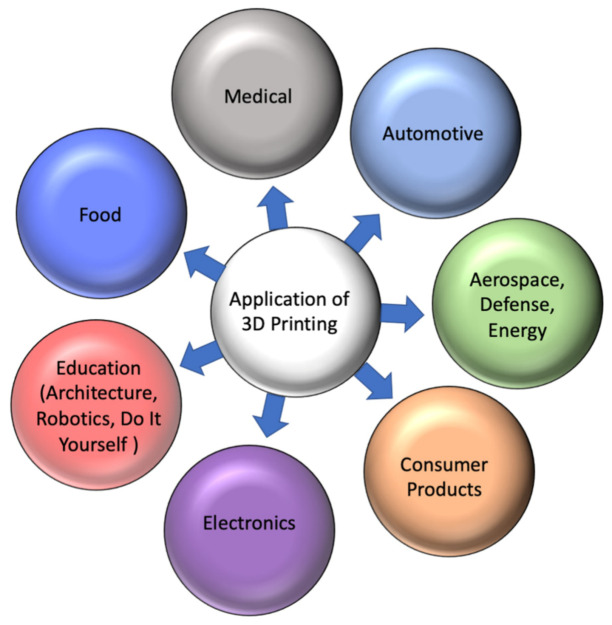
Typical current and possible future application of 3D printing technologies.

With improving properties and new AM techniques coming up lately, it has become possible to take the 3D printing manufacturing approach to its next level. Three-dimensional printing technology has been useful in various and varied areas. Figure 12 shows the various kinds of usages of 3D printing which also comprise applications in research and development, visual aids, presentation models, artistic items, device covers, custom parts, functional models, and patterns together with series production. Injection molding 3DP has also become the most used manufacturing process for the automated fabrication of plastic moldings. By applying this advanced printing, plastic parts with complex geometries are being manufactured quickly and cost-efficiently without any significant amount of postprocessing. Prototyping of different components in aerospace industries has always been the key to developing new lightweight products, which is why 3D printing of late has made sufficient inroads into this industry. In addition, 3DP offers possibilities of waste reduction and emissions in the aerospace industry. AM has been primarily used in military applications so far. However, it would be highly beneficial to deploy this technology in the arena of the commercial aerospace industry.

AM printing could offer a novel approach in not only producing the desired customer-customized parts, such as engine blocks, but also drastically reducing the manufacturing costs for assemblies, particularly for the automotive sector. The automotive industry can significantly gain from the 3D printing technologies in multiple ways. Firstly, the size of the single car components can be considerably bigger as 3D printers can build more complex structures. Car parts could be printed directly where needed (in the car). Additionally, AM could condense spare part inventories since advanced printing has the capability to manufacture spare parts on the customer’s demand. So, only the needed raw materials must be maintained in stock. Like this, 3DP could enable a large cost-saving perspective by removing the requirement for large spare part inventories.

**Figure 13:**
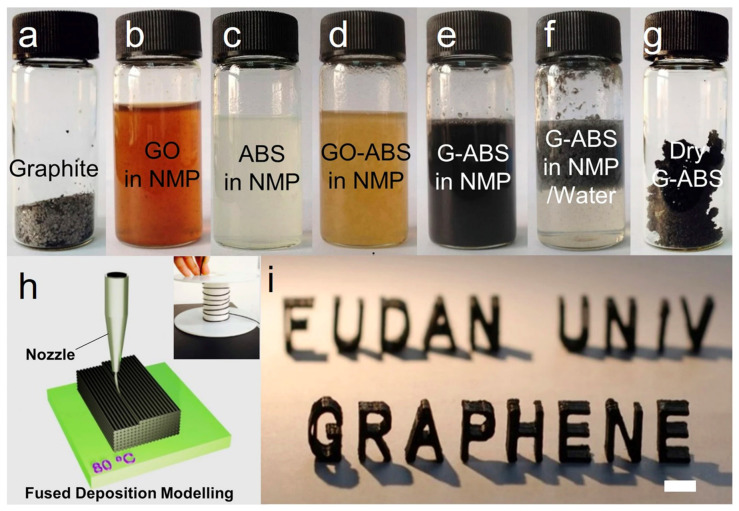
Photograph of (**a**) graphite flakes; (**b**,**c**) dispersions of graphene oxide(GO) and ABS in N-methylpyrolidone (NMP) solvent; (**d**,**e**) a homogeneous mixture of GO-ABS in NMP before and after chemical reduction; (**f**) graphene(G)-ABS clots found after isolation (**e**) with water; (**g**) G-ABS composite powder after washing and drying; (**h**) schematic illustration of fused deposition modeling 3DP, the inset shows the graphene-based filament winding on a roller; (**i**) a typical 3D printed model consuming 3.8 wt% G-ABS composite filament, scale bar: 1 cm. Reprinted with permission from reference [104], Copyright 2015, Nature.

Until lately, 3D printing was used mostly for prototyping reasons in the footwear and food industries. However, conventional market players together with emerging startups have started investing in AM technologies in recent years. The promising outcomes have been 3D printing of nanocomposites for various electrical and electronics applications. Wei et al. [104] confirmed that, a graphene reinforced acrylonitrile-butadiene-styrene (ABS) composite could be fused deposition modelling (FDM) printed into computer-designed models (Figure 13) and improved electrical conductivity was detected.

**Figure 14:**
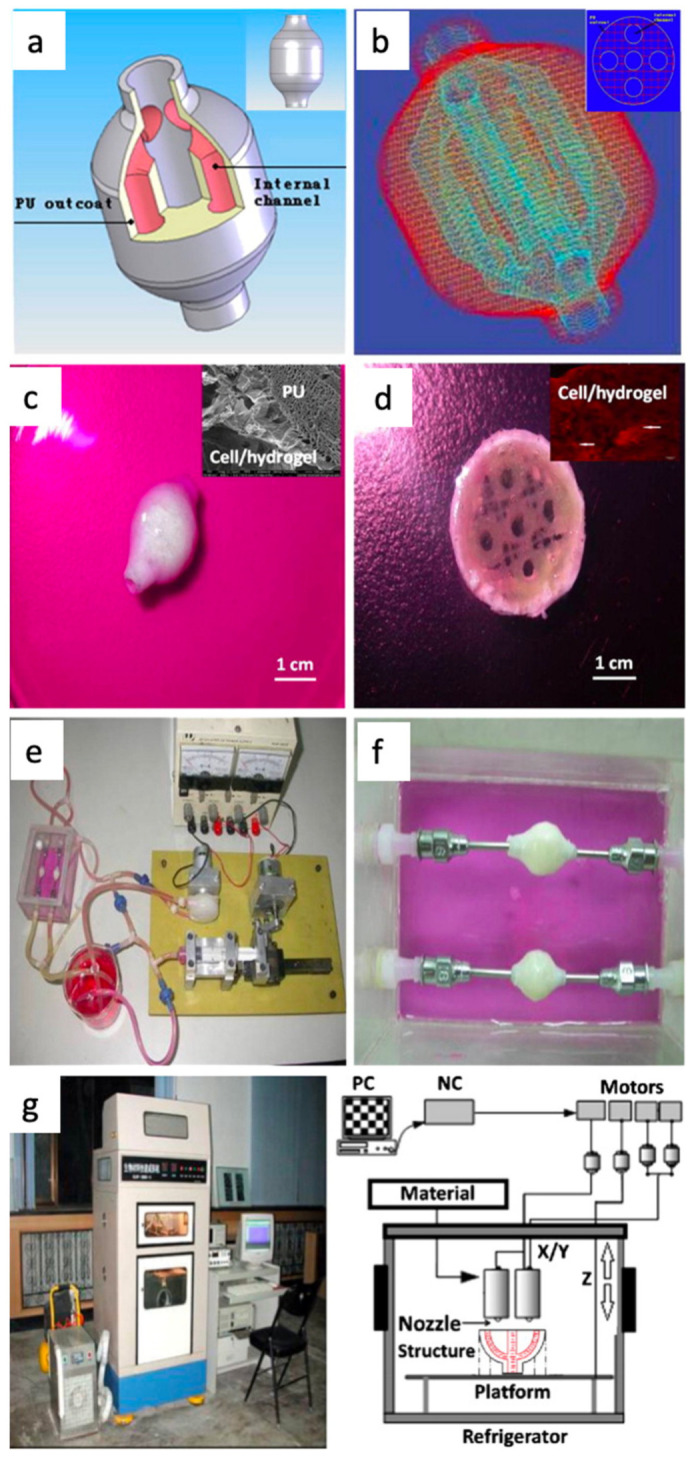
A sizable scaled-up 3DP-driven complex organ with vascularized liver tissue construct using a double-nozzle 3PD set-up built in Tsinghua University, Prof. Wang’s laboratory. Representation of the hybrid hierarchical construct fabricated via a DLDM RP system: (**a**) a digital CAD model with an outlook (the inset image) and an internal branched network; (**b**) a common layer interface (CLI) file and a cross-sectional vision (the inset image) of the CAD model; (**c**) a DLDM product with a PU outcoat; the inset image displays the SEM result of the in vitro cultured sample with porous PU and cell/hydrogel layers; (**d**) the middle part of (**c**) with branched/grid internal cell/hydrogel channels; the inset image displays the PI staining result of the cell/hydrogel section; (**e**) two complex constructs undergoing in vitro pulsatile culture; (**f**) a magnified view of (**e**); (**g**) the DLDM RP device and a schematic design of the printing processes; cells were loaded in the natural gelatin/alginate/fibrinogen hydrogel and deposited with one nozzle while synthetic PU was added with another nozzle. Images reproduced with permission from reference [106], Copyright 2013, with permission from Elsevier.

Three-dimensional printing also holds the potential to unscramble limitations in existing biomedical industries. It can be expanded to rapidly manufacture personalized tissue engineering scaffolds, in situ restoration of damaged tissues within cells, and even straightway print tissue and organs. Recently, Wang et al. [105] have reported, a hybrid hierarchical polyurethane (PU)–cell/hydrogel construct that was automatically shaped exploiting an extrusion-based double-nozzle and low-temperature 3D printer (Figure 14a–g).

There are two general trends that are expected to reshape the future of the AM technology: (1) switching from rapid prototyping to rapid manufacturing and (2) the option to enable mass customization of end-products using 3DP.

**Figure 15:**
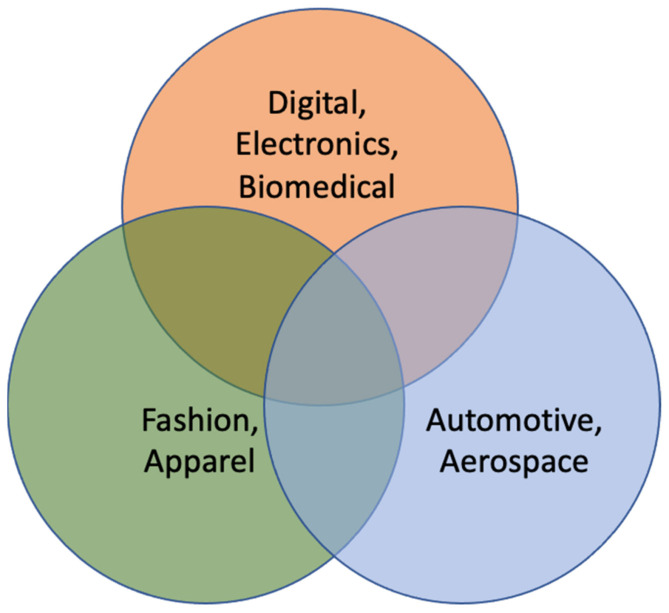
Present and future applications of 4D printing in different sectors of industry.

As has already been explained, 4D printing, being an upcoming advanced version of the existing 3DP technology, is well poised to help accelerate growth in many research and industrial sectors. Some of the potential sectors include aerospace, engineering, automobiles, energy, manufacturing, defense, medicine and various other fields. Four-dimensional printing offers better reliability, improved consistency, and superior performance characteristics over 3D printing. Initial types of 4D printers contain versatile 3D structures pre-printed via 3D printing and that can be transformed to other sizes and shapes when immersed in water or exposed to light, pressure, gravity, or air [107]. Figure 15 summarizes the important roles of 4D printing in the field of manufacturing, as applicable to different industrial sectors. Although 4D printing has been in the manufacturing market over the last few years, its supply chain, with faster shipping and lower storage space options offer definite advantages. Four-dimensional printing removes a large degree of transportation and processing expenses, thus providing the supply chain with incredible applications [108,109].

**Figure 16:**
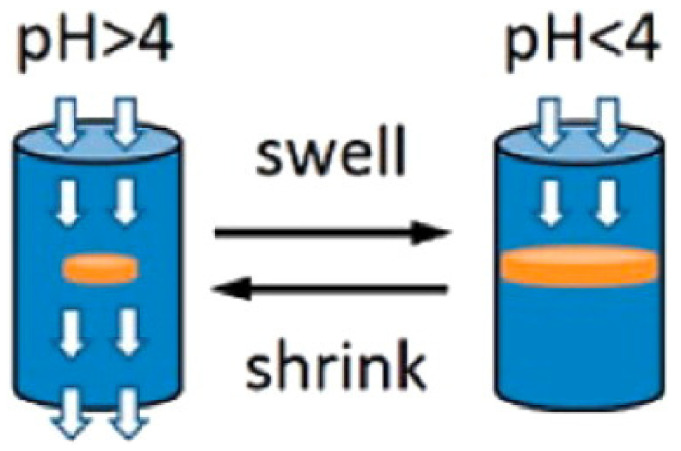
pH-responsive flow regulating smart valve. Reprinted from reference [110], with the permission of American Chemical Society, Copyright 2016.

**Figure 17:**
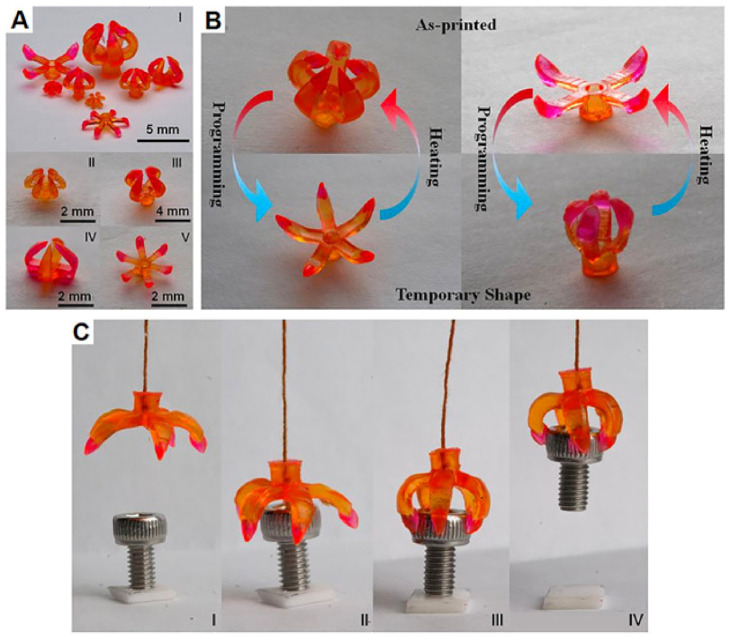
Grippers printed using 4DP as biorobots. (**A**) Multimaterial grippers of distinctive designs; (**B**) validation of the transition between as printed and brief shapes of multimaterial grippers; and (**C**) photographs of the gripping activity to clutching an object. Reproduced with permission from reference [73] Copyright 2016, Nature.

Four-dimensional printing grants the freedom to change its shape over time to 3D printed parts and objects. Printed components and structures could change shape due to a difference in temperature, air pressure, pH conditions, or other environmental changes. Figure 16 shows a 4D printed pH-responsive flow regulating smart valves. Four-dimensional technology can easily replenish hinges or even hydraulic motors and drives, making mechanical gears easier and smaller. Robotics is yet another area of application where 4D printing has proven its mettle. Figure 17A–C describe a 4D printed grippers and their use as biorobots.

**Figure 18:**
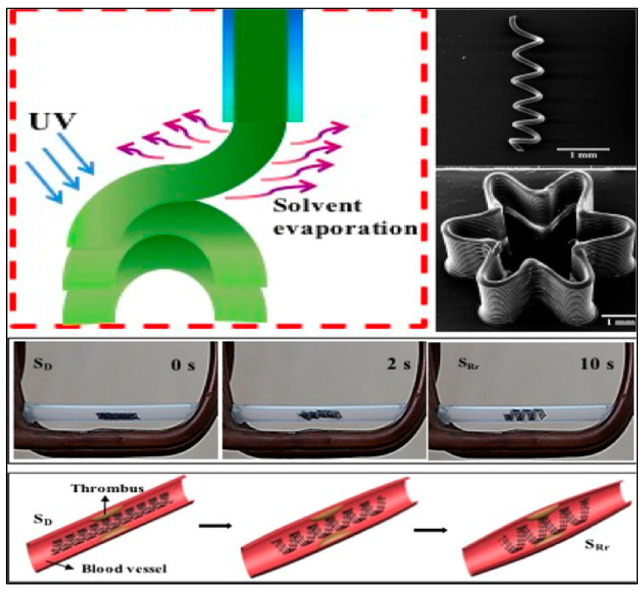
Direct ink writing (DIW) 4D printing showing printing mechanism, bending in presence of magnetic field, and application as intravascular stents. Reproduced from Ref. [111] with permission. Copyright 2017, American Chemical Society.

Very encouraging applications of 4D printing in the field of medicine have been reported. One such example is the 4D printing of intravascular stents. The entire research work has been captured in a diagrammatic representation (Figure 18) where the printing process, along with the bending in the presence of a magnetic field, and intravascular stent application has been explored. Four-dimensional printing is offering benefits to both doctors and researchers, alike, particularly in areas not obscured by the 3D printing technologies. This printing technology can deliver extensive support in the biomedical field, especially with better and smart biomedical implants, tools, sensors, actuators, and devices. It is hoped that the medical practitioners will be able to navigate 4DP technology to serve their patients better.

The complex assemblies in the various applications will be way too labor-intensive and costly to manufacture using conventional fabrications technologies. The beauty of advanced manufacturing and printings lies in utilizing. Both the 3D and 4D printing offer great advancements, on the current standards, and are incredible technologies for the consumers, 4DP is more advanced, focusing futuristic applications on the technologies of the 3DP. However, 4D printing technology can have applications throughout numerous sectors as research and developmental efforts progress.

**Figure 19:**
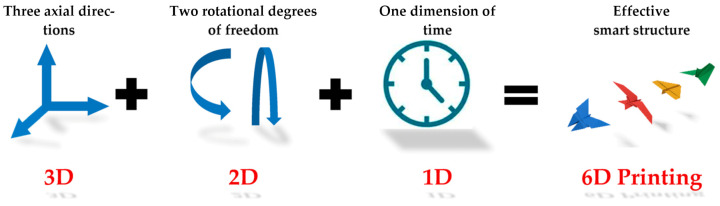
Concept of 6D printing. Reprinted with permission from reference [115], Copyright 2021 by the authors and license MDPI, Basel, Switzerland.

However, the notion of 5D printing is being talked about now and even investigated with [112]. Five-dimensional printing is not just an advanced version of 3D or 4D printing; it is a new branch of advanced printing. In this printing process, the printable object and the printhead have five degrees of freedom. In its place of the regular layer, it manufactures complex curved layers. In this AM process, the print object moves while the head of the printer is printing [113,114]. Thus, printing commences the curve path of the object being printed rather than moving through a traditional level as in the case of what usually happens for 3D printings. The main advantage of this 5D printing process is to produce a part with a curved layer with better mechanical strength. Added major advantage of this new advanced manufacturing technology is it uses less material compared to 3D printing. The new printing technique which combines the four-dimensional and five-dimensional printing techniques has also been conceptualized (Figure 19). This means that the new six-dimensional (6D) printing is going to utilize five degrees of freedom and the final manufactured component will be a smart object [115,116]. This will be qualified for changing its properties or shape owing to its interaction with an external stimulus. A 6D printed object can be manufactured using less material, can perform movements by being exposed to an outer stimulus, and can be stronger than an equivalent 4D printed structure. Six-dimensional printings can allow the printed object to learn how to reconfigure itself appropriately, based on predictions via mathematical modeling, artificial intelligence, and external simulations.

## 5. Conclusions

While 3D printing of metal, ceramic and polymer composite materials has undergone substantial expansion in recent years, it is still not extensively recognized by most manufacturing industries. Some of the limitations that have hindered the accelerated market penetrability include materials availability and compatibility, unreliable mechanical performances of the printed 3D objects and parts, and the time-consuming printing process. Even with these constraints, 3DP methods, in recent years, have registered impressive growth patterns in aerospace, automotive, energy and medicine. It has been evidenced that researchers are studying new materials and new applications relating to 3D printing technologies. Four-dimensional printing, on the other hand, offers some definite advantages over the existing 3D printing methods and has shown its potential applications on technologies that could be done by the 3DP. In the aerospace, automotive, defense, energy and medical areas, smart materials such as piezoelectric and shape-changing materials are already being used, albeit on a smaller scale. This advanced manufacturing technology can manufacture programmable smart materials capable of changing shape and functional properties by means of external environmental stimuli. This could be the basis for intelligent biomedical devices, implants, precision medicine, and programmable tissues that could be helpful to medical practitioners to serve better for particular applications. However, in 4D printing, there are still few difficulties and challenges, such as high technology cost, lack of efficient supply chain and materials restrictions in mechanical properties of materials and control of physical changes, which need to be addressed. Researchers, globally, are working at a fever pitch to surmount these challenges by pursuing intense research and developmental activities.

## Figures and Tables

**Table 1:** Characteristic distinctions between 3D and 4D printing techniques.

3DP	4DP	Compared Characteristics
Structure formed using layer-by-layer deposition of 2D ink material	One more step of advancement of 3D printing with shape-changing programming property	Manufacturing approach
Metals, ceramics, thermoplastics, nano-, and biomaterials,	Smart materials	Printable materials
No programming step involved	Thermomechanical preparation, multimaterial printing to create differential thermal and mechanical stresses	Shape-memory programming
Forms rigid structure	Characteristics of structure flexibility change upon exposure of external energy stimulus	Shape flexibility
Engineering, electronics, medicine, dentistry, automotive, robotics, fashion, aerospace, defense, and nuclear etc.	Adds dynamic element(s) to all 3D printing applications, mostly employed in biomedical industries	Area of applications

**Table 2:** Comparison of the widely used different types of 3D printing technologies.

	Fused Deposition Modeling (FDM)	Stereolithography (SLS)	Selective Laser Sintering (SLA)
Materials	Thermoplastic	Photo-active polymers	Powders of metal, alloys
Time of manufacturing	Fast	Medium	Low
Cost of manufacturing	Low	Medium	Very high
Printing accuracy	Low	Medium	High
Application	Appropriate for printing prototypes for home use	Exceptional for printing of water-resistant material	Perfect for printing functional parts with numerous applications based on complex shape and heat and chemical resistant materials

**Table 3:** Summary of advanced material strategies for additive manufacturing.

Material Category	Materials	Advanced Printing Technique	Applications
Smart	Piezoelectric Shape-changing	4D printing, material extrusion, material jetting, EHD printing, directed energy deposition, vat photopolymerization, 2-photon photolithography, digital light processing, stereolithography	Biomedical devices, soft microrobotics, drug delivery vehicles, piezoelectric devices
